# Acquisition of RGC phenotype in human Müller glia with stem cell characteristics is accompanied by upregulation of functional nicotinic acetylcholine receptors

**Published:** 2013-09-13

**Authors:** Silke Becker, Shweta Singhal, Megan F. Jones, Karen Eastlake, Phillippa B. Cottrill, Hari Jayaram, G. Astrid Limb

**Affiliations:** 1 Division of Ocular Biology & Therapeutics, Institute of Ophthalmology, University College London, London, UK; 2 NIHR Biomedical Research Centre for Ophthalmology, UCL Institute of Ophthalmology and Moorfields Eye Hospital, London, UK

## Abstract

**Purpose:**

Human Müller glia with stem cell characteristics (hMGSCs) can be induced to express genes and proteins of retinal ganglion cells (RGCs) upon in vitro inhibition of Notch-1 activity. However, it is not known whether expression of these markers is accompanied by acquisition of RGC function. This study investigated whether hMGSCs that express RGC markers also display neural functionality, as measured by their intracellular calcium concentration ([Ca^2+^]_i_) responsiveness following neurotransmitter stimulation in vitro*.*

**Methods:**

Changes in mRNA expression of RGC markers and neurotransmitter receptors were assessed either by conventional or quantitative reverse transcription PCR (RT-PCR), while changes in protein levels were confirmed by immunocytochemistry. The [Ca^2+^]_i_ levels were estimated by fluorescence microscopy.

**Results:**

We showed that while undifferentiated hMGSCs displayed a profound elevation of [Ca^2+^]_i_ after stimulation with N-methyl-D-aspartate (NMDA), this was lost following Notch-1 inhibition. Conversely, untreated hMGSCs did not respond to muscarinic receptor stimulation, whereas [Ca^2+^]_i_ was increased in differentiated hMGSCs that expressed RGC precursor markers. Differentiated hMGSC-derived RGCs, but not undifferentiated hMGSCs, responded to stimulation by nicotine with a substantial rise in [Ca^2+^]_i_, which was inhibited by the α4β2 and α6β2 nicotinic receptor antagonist methyllycaconitine. Notch-1 attenuation not only caused a decrease in the gene expression of the Notch effector HES1 and increased expression of RGC markers, but also an increase in the gene and protein expression of α4 and α6 nicotinic receptor subunits.

**Conclusions:**

These observations suggest that in response to Notch-1 inhibition, hMGSCs differentiate into a population of RGCs that exhibit some of the functionality observed in differentiated RGCs.

## Introduction

The presence of Müller glia with stem cell characteristics has been reported in a range of mammalian species, including the adult human retina [1-5]. Evidence for the regenerative ability of Müller glia has been demonstrated in postnatal chick [6] and rat retinas [7], and compelling proof for the neurogenic properties of these cells was shown in the zebrafish, in which Müller glia can dedifferentiate, reenter the cell cycle, and differentiate into retinal neurons [8-10]. Although Müller glia with stem cell characteristics (hMGSCs) are present in the adult human retina [11], they appear to have lost their regenerative ability in situ. However, they can be isolated and grown indefinitely in culture [12] and retain their capacity to differentiate into retinal neurons in vitro in response to factors identified during development [5]. This makes hMGSCs strong candidates for cell replacement strategies in retinal degenerative conditions characterized by neural cell damage.

Previous investigations have demonstrated that hMGSCs concomitantly display various features of neural stem cells and retinal progenitors, such as the expression of *SOX2*, *PAX6*, and *CHX10*. The presence of markers of differentiated retinal neurons strongly indicates that hMGSCs represent retinal progenitors with the capacity to form retinal neuronal cells [5], including retinal ganglion cells (RGCs) [13], the cell type lost in glaucoma. Notch-1, which controls the development of RGCs in the embryonic retina [14] and which is present in low quantities in Müller glia of healthy retina [15], has been shown to be highly expressed in hMGSCs [5]. We have previously demonstrated that Notch-1 inhibition in hMGSCs, using the γ−secretase inhibitor N-[N-(3,5-Difluorophenacetyl)-L-alanyl]-S-phenylglycine t-butyl ester (DAPT), induces these cells to acquire neural morphology and to express gene and proteins characteristic of RGC precursors, including BRN3B, *ISL-1*, and *HUD* [13].

Neural progenitors of the central nervous system express receptors for various neurotransmitters [16,17], which upon binding to ligands induce changes in the membrane potential [18]. However, expression of these receptors is not confined to neurons, and changes in membrane potential do not necessarily lead to a rise in cytosolic calcium ([Ca^2+^]_i_), which has been progressively accepted as indicative of neuronal cell function [18,19]. While some neurotransmitter receptors have been identified in neural progenitors, others are exclusively expressed in differentiated neurons [20], providing a tool for the identification of the maturation stages of neural cells. At the time of optic cup formation, neural retinal progenitors in the ventricular zone express receptors for muscarinic, purinergic, γ-aminobutyric acid (GABA), and glutamatergic systems [20]. These are thought to play a role in the differentiation of retinal progenitors [21] and their differentiation and function can be used as indicators of retinal neural differentiation.

**Figure 1:**
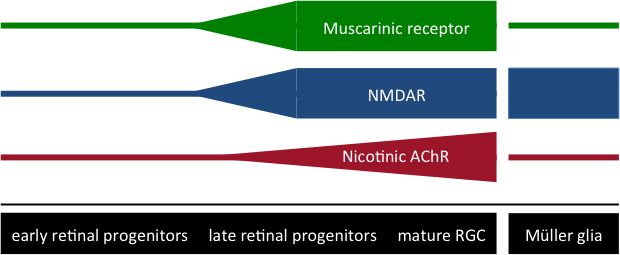
Expression levels of neurotransmitter receptors differ in early and late retinal progenitors, as well as in Müller glia. Varying expression levels of N-methyl-D-aspartate (NMDA) receptors, muscarinic receptors and nicotinic acetylcholine receptors (AChR) are depicted throughout development in early and late retinal progenitors and mature retinal ganglion cells (RGCs), as well as in Müller glia.

The nicotinic, glutamatergic, and muscarinic receptor-ligand systems play a significant role in RGC development [22,23]. Since their expression changes throughout various stages of RGC differentiation, they can be examined to identify whether acquisition of markers of RGC-committed precursors by differentiated Müller stem cells is accompanied by expression of RGC functionality. In particular, the expression of nicotinic acetylcholine receptors (nAChR), which are present in retinal stem cells and early retinal progenitors, is greatly upregulated in late retinal progenitors [20]. The expression of different nAChR subunits is likely to be differentially regulated throughout development [22]. Conversely, functional expression of N-methyl-D-aspartate (NMDA) receptors is highest in late retinal precursors [19,22,23] and in mature RGCs [24] , as well as in Müller glia cells [25], but not early retinal precursors [20]. Muscarinic receptors, which are only sparsely expressed in early retinal progenitors and Müller glia cells, have been shown to be abundantly expressed in late retinal progenitors [20,26] (Figure 1).

Although these neurotransmitter receptors are also expressed by Müller glia [20,25,27], changes in levels of expression of these molecules by hMGSCs may indicate acquisition of neural function and can be used to estimate the ontogenetic stage of the retinal precursors generated. On this basis, we investigated whether downregulation of Notch-1 in hMGSCs, in addition to inducing phenotypic changes characteristic of RGCs, also leads to neural functionality as judged by an increase in [Ca^2+^]_i_ in response to selective neurotransmitter stimulation.

## Methods

### Culture of Müller glia with stem cell characteristics

An hMGSC line derived in our laboratory and known as MIO-M1 was maintained for up to 40 passages in Dulbecco’s Modified Eagle Medium (DMEM, 1× with GlutaMAX™, without sodium pyruvate; Gibco, Life Technologies, Carlsbad, CA or DMEM high glucose™, PAA laboratories, Pasching, Austria), supplemented with 10% fetal calf serum (FCS, PAA laboratories) as well as 20 U/ml penicillin and 20 μg/ml streptomycin (Gibco, Life Technologies). To passage cells, confluent monolayers were usually detached once a week using TrypLE™Express (Gibco, Life Technologies) and subcultured at a dilution of 1:5 to 1:6.

### Differentiation of human Müller glia with stem cell characteristics towards procursors committed to an RGC fate

Differentiation of MIO-M1 cells into RGC precursors was induced as previously described [13] by culturing cells for 7 days on surfaces coated with 0.5 µg/ml basement membrane protein (BMP, ECM gel from Engelbreth-Holm-Swarm murine sarcoma, Sigma-Aldrich, St. Louis, MO) with 20 ng/ml basic fibroblast growth factor-2 (FGF2, Sigma-Aldrich) in the absence or presence of 50 µM DAPT (Sigma-Aldrich, St. Louis, MO). MIO-M1 cells cultured in the absence of these factors were used as controls.

### Assessment of cytosolic Ca^2+^ following neurotransmitter stimulation

MIO-M1 cells were grown for 7 days on LAB-TEK™ 8-well chambered coverglasses (Nalge Nunc™, Rochester, NY) to approximately 60% confluency. Cells were divided into three treatment groups, which received either no treatment (control) or which were cultured on BMP-coated surfaces with 20 ng/ml FGF2 in the absence (BMP/FGF2) or presence of 50 µM DAPT (BMP/FGF2/DAPT). Cells were loaded with Fura Red-AM (2 µg/ml, Invitrogen, Life Technologies) in serum-free DMEM for 30 min at 37 °C. Before stimulation with neurotransmitters, cells were serum-recovered in DMEM supplemented with 10% FCS for at least 30 min at 37 °C, to allow for deesterification of the dye, and were subsequently transferred into 200 µl phenol-red free Leibovitz’s medium L-15 (Gibco, Life Technologies). Inhibitors of nicotinic acetylcholine receptors (100 nmoles/l methyllycaconitine citrate hydrate (MLA), Sigma-Aldrich, or 100 nmoles/l α-conotoxin MII (α-CT), Tocris, Bristol, UK) were added in Leibovitz’s medium L-15 (Gibco, Life Technologies) at least 15 min before the addition of nicotine.

Cells were transferred onto the stage of a Leica TCS-SP2 inverted microscope (Leica, Wetzlar, Germany) and kept at 37 °C inside a heated stage chamber. Cells were exposed to excitation light at a wavelength of 488 nm, and emission light of 550–600 nm was recorded. Images were taken at 20× magnification at a frequency of 2 Hz for 75 s and were stored on a PC for off-line analysis. All agonists were dissolved in water (ddH_2_O) at a concentration of 10 mM, and 50 µl of this solution were added to the bath to achieve a final concentration of 2 mM. Fluorescence intensity during the recording period was measured with LCS Lite software individually for each cell with background subtraction, with the inverted fluorescence intensity used as an estimate of [Ca^2+^]_i_. Changes in [Ca^2+^]_i_ were determined as the percentage of the [Ca^2+^]_i_ at rest and were averaged for all cells recorded in the experiment. Data are displayed as average [Ca^2+^]_i_ ± SEM in n separate experiments. The rise in [Ca^2+^]_i_ was validated by exposure of untreated and differentiated MIO-M1 cells to 2 mM histamine (n=3), which caused a profound reduction in the fluorescence intensity, corresponding to a substantial sustained rise in [Ca^2+^]_i_ (data not shown). The fraction of cells responding to agonist stimulation with a rise in [Ca^2+^]_i_ (“responders”) was determined as a percentage of the total number of cells in the field, and is displayed as mean ± SEM.

### RNA isolation, reverse transcription and PCR

Cells were grown to approximately 90% confluency in DMEM or DMEM supplemented with growth and differentiation factors (see above), detached with TrypLE™Express, washed in phosphate buffered saline (PBS), and pelleted at 4 °C. Total RNA was extracted using the RNeasy Mini Kit (Qiagen, Hilden, Germany), including DNase treatment according to the manufacturer’s instructions. The amount of 2 µg RNA was reverse transcribed using the First Strand cDNA Synthesis kit (0.5 µg RNA/10 µl reaction, Roche Applied Science, Penzberg, Germany). The reaction mixture was incubated for 10 min at 25 °C, 60 min at 42 °C, 5 min at 99 °C, and 5 min at 4 °C in a Mastercycler gradient thermal cycler (Eppendorf, Hamburg, Germany).

PCR was subsequently performed using the Expand High Fidelity PLUS PCR System (Roche Applied Science) with specific primers for *BRN3B, HES1*, nAChR α subunits 1–4, 6, and 7, β-actin and *GAPDH* (Invitrogen, Life Technologies, see Table 1). PCR mixtures were incubated at 94 °C for 2 min, followed by 24 to 40 cycles of 94 °C for 30 s, 58 °C for 30 s, 72 °C for 1 min and finally held at 72 °C for 5 min. PCR products were analyzed by electrophoresis on 1% agarose gel. Band density was quantified by densitometry using ImageJ software, and results were expressed as the ratio of the band density of the target gene over that of β-actin or *GAPDH* as the housekeeping gene.

**Table 1:** PCR primers

Genes	Sequence	Product size (bp)	Ref.
BRN3B	CAGGTTCGAGTCCCTCACAC	198	Primer bank id: 4758948a2
	ATGGCAAAGTAGGCTTCGAGC		
HES1	AAGATAGCTCGCGGCATTCCA	160	Primer-BLAST
	CGTTCATGCACTCGCTGAAG		
nAChRα1	GCTCTGTCGTGGCCATCAA	70	[45]
	CACTCCCCGCTCTCCATG		
nAChRα2	CTCCCATCCTGCTTTCCAG	115	[46]
	GTTTGAACAGGCGGTCCTC		
nAChRα3	AACCTGTGGCTCAAGCAAATCT	78	[45]
	CATGAACTCTGCCCCACCAT		
nAChRα4	GTGGATGAGAAGAACCAGATGATG	74	[45]
	CAGCGCAGCTTGTAGTCGTG		
nAChRα6	TGGCCAACGTGGATGAAGTAA	111	[45]
	TCTCAATGCCATCATATTCCATTG		
nAChRα7	GCCAATGACTCGCAACCACTC	335	[47]
	CCAGCGTACATCGATGTAGCA		
β−αχτιν	CACTCTTCCAGCCTTCCTTC	314	[48]
	CTCGTCATACTCCTGCTTGC		
GAPDH	CCAGTGCAAAGAGCCCAAAC	225	Primer bank id: 2282013a2
	GCACGGACACTCACAATGTTC		

Prior to running quantitative RT-PCR reactions, primer concentrations and amplification conditions were optimized and the presence of a single PCR product was confirmed by agarose gel electrophoresis. The efficiency of PCR product amplification was assumed to be 100%. Quantitative RT-PCR reactions were subsequently performed using SYBR Green JumpStart Taq ReadyMix (Sigma-Aldrich) according to the manufacturer’s instructions with 0.4 pmoles/l primers and 2 μl cDNA in a total volume of 25 μl. Twelve repeats were prepared for each experimental condition and transferred to an AB 7900HT Real-Time PCR System (Applied Biosystems, Life Technologies). Quantitative RT-PCR mixtures were incubated at 94 °C for 2 min, followed by 40 cycles of 94 °C for 15 s, 60 °C for 30 s, and 72 °C for 1 min as well as a dissociation stage with a SYBR green detector for quality control of the PCR product. Data analysis was performed using DART-PCR version 1.0 software. The relative expression values (R_0_) of the test genes were normalized to the R_0_ values of the housekeeping gene, and the ratios obtained were compared as the fold change in mRNA expression.

### Immunocytochemistry

MIO-M1 cells were grown for 7 days in DMEM supplemented with 2% FCS on BMP-coated glass coverslips in 24-well plates and divided into two treatment groups, which received either no treatment (control) or treatment with 20 ng/ml FGF2 and 50 µM DAPT (BMP/FGF2/DAPT). After 7 days cells were fixed in 4% paraformaldehyde for 10 min, cryoprotected using 30% sucrose and stored at −20 °C until required. After defrosting, slides were blocked for 1 h at room temperature using either Roche blocker (0.5% Blocking Solution, Roche Applied Science, with 5% donkey serum) or Tris-buffered saline (TBS) with 0.3% triton and 5% donkey serum. Primary antibodies (see Table 2) were diluted in blocking serum and incubated overnight at 4 °C. Primary antibody labeling was detected using donkey antigoat or antimouse antibodies labeled with Alexa Fluor 488 (1:500, Molecular Probes, Invitrogen) for 3 h at room temperature. 4',6-diamidino-2-phenylindole (DAPI; 1 μg/ml, Sigma-Aldrich) was used to counterstain the cell nuclei, and slides were mounted with Vectashield (Vectashield, Vector Laboratories, Burlingame, CA). Fluorescent images were captured with identical exposure times using a Zeiss LSM710 confocal microscope and identically processed using Carl Zeiss Zen imaging software (Carl Zeiss Microscopy GmbH, Jena, Germany).

**Table 2:** Antibodies for immunocytochemistry

Primary antibody	Source	Host	Dilution
Vimentin	Santa Cruz Biotech (sc-5565)	Rabbit	1:100
CRALBP	Santa Cruz Biotech (sc-28193)	rabbit	1:100
Isl-1	Hybridoma Bank (39.4D5)	Mouse	1:100
βIII-tubulin	Millipore MAB1637	Mouse	1:100
THY-1(CD90)	BioLegend (328,106)	biotinylated	1:100
AChR α4	Santa Cruz Biotech (sc-74519)	Mouse	1:50
AChR α6	Santa Cruz Biotech (sc-27292)	Goat	1:50

### Statistical analysis

Results are expressed as means ± standard error of the mean (SEM), and statistical significance was determined by one-way repeated measures ANOVA with differences considered to be significant for p<0.05.

## Results

### Expression of RGC precursor markers upon Notch-1 inhibition in hMGSCs

**Figure 2:**
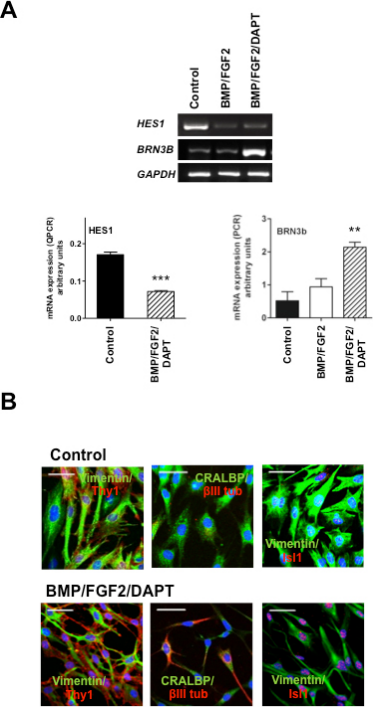
Expression of retinal ganglion cell (RGC) markers following Notch-1 inhibition in human Müller glia with stem cell characteristics (hMGSCs) is consistent with the acquisition of a neural phenotype. **A**: Inhibition of Notch-1 by treatment with basement membrane protein (BMP), basic fibroblast growth factor-2 (FGF2) and N-[N-(3,5-Difluorophenacetyl)-L-alanyl]-S-phenylglycin t-butyl ester (DAPT) attenuated mRNA expression of *HES1* (***p<0.001, n=4), while significantly upregulating *BRN3b* mRNA, a marker of RGC precursors (**p<0.01, n=4), as shown by quantitative and conventional reverse transcription PCR (RT-PCR), respectively. **B**: In untreated hMGSCs, the Müller cell markers vimentin and CRALBP (green) were highly expressed, while THY1, βIII-tubulin, and ISL-1 (red), which are characteristic of RGCs, were undetectable or found at low levels (upper panel). After differentiation of hMGSCs by Notch-1 inhibition, the expression of vimentin and CRALBP (green) was attenuated, while that of THY1, βIII-tubulin, and ISL-1 was augmented (red, lower panel).

Acquisition of RGC precursor markers in hMGSCs triggered by the attenuation of Notch-1 activity was confirmed using RT-PCR and quantitative RT-PCR. Figure 2A illustrates that treatment of hMGSCs with FGF2 and DAPT on BMP-coated surfaces induced strong mRNA downregulation of the Notch-1 effector *HES1* (***p<0.001) and upregulation of the RGC marker *BRN3B* (**p<0.01), as demonstrated by quantitative and conventional RT-PCR, respectively. Ct values for quantitative RT-PCR of *HES1* are shown in Table 3.

**Table 3:** Cycle threshold values for quantitative RT–PCR of *HES1*

Experimental condition	Cycle threshold (Ct)
Control	25.83±0.05
BMP/FGF2/DAPT	25.09±0.06

As shown in Figure 2B immunocytochemical staining demonstrated high intracellular expression of the Müller cell markers vimentin and CRALBP in undifferentiated hMGSCs, while the RGC and neuronal markers THY-1, βIII-tubulin, and ISL-1 were not detectable or expressed at very low levels (upper panel). Conversely, after differentiation of hMGSCs by Notch-1 inhibition using BMP, FGF2, and DAPT, protein expression of vimentin and CRALBP was greatly attenuated, while THY1, βIII-tubulin, and ISL-1 were shown to be strongly upregulated (lower panel), indicative of a change from a glial to a retinal neuronal phenotype.

### Effect of Notch-1 inhibition on hMGSCs on cytosolic [Ca^2+^]_i_ levels in response to NMDA receptor activation

**Figure 3:**
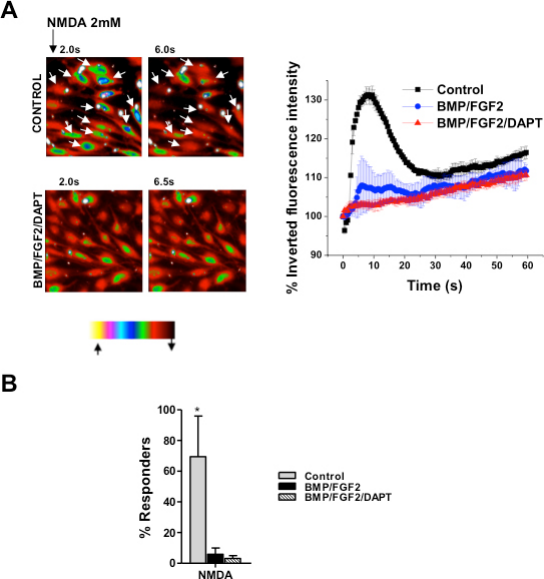
Cytosolic calcium ([Ca^2+^]_i_ ) response to N-methyl-D-aspartate (NMDA) receptor activation is consistent with the development of retinal ganglion cell (RGC) phenotype by Notch-1 inhibition in human Müller glia with stem cell characteristics (hMGSCs). **A**: Examplary heat map images are shown with Fura Red-loaded hMGSCs cultured under control conditions and after treatment with basement membrane protein (BMP), basic fibroblast growth factor-2 (FGF2) and N-[N-(3,5-Difluorophenacetyl)-L-alanyl]-S-phenylglycin t-butyl ester (DAPT). The images are recorded at 40× magnification and are representative of fluorescence intensity before (2 s) and at the maximum effect of receptor activation (6 s and 6.5 s, respectively). The color bar describes the intensity–color relationship, with yellow being the brightest and black being the dimmest. Control cells showing a rapid decrease in fluorescence intensity in response to NMDA exposure, which signifies an increase in [Ca^2+^]_i_, are marked with white arrows. Cells cultured with BMP, FGF2, and DAPT did not respond to NMDA with a reduction in fluorescence intensity (left panel). In response to NMDA (2 mM), untreated hMGSCs (control, n=41 cells from two experiments) showed a strong reduction in the inverted fluorescence intensity, displayed as a percentage of the value at 0 s, corresponding to an increase in [Ca^2+^]_i,_. This was absent in hMGSCs treated with either BMP or FGF2 alone (n=32 cells from two experiments) or a combination of BMP, FGF2, and DAPT (n=50 cells from 3 experiments), *** p<0.001 control versus BMP/FGF2 or BMP/FGF2/DAPT (right panel). **B**: The fraction of cells responding to NMDA with a rise in [Ca^2+^]_i_ was greatly diminished after Notch-1 inhibition following differentiation without or with DAPT, in comparison to control cells (n=50 from four experiments, respectively, *p<0.05).

Figure 3A shows that following Notch-1 inhibition in hMGSCs, the average rise in [Ca^2+^]_i_ in response to NMDA receptor stimulation was largely decreased, compared with untreated cells (***p<0.001). There was no significant difference in the average [Ca^2+^]_i_ response for cells treated with BMP and FGF2 alone or in the presence of DAPT. As demonstrated in Figure 3B, the proportion of cells responsive to NMDA stimulation was greatly reduced after Notch-1 inhibition (*p<0.05). There was no significant difference in the percentage of cells responsive to NMDA for cells treated with BMP and FGF2 alone or in the presence of DAPT**.**

### Effect of Notch-1 inhibition in hMGSCs on cytosolic [Ca^2+^] levels in response to muscarinic receptor activation

**Figure 4:**
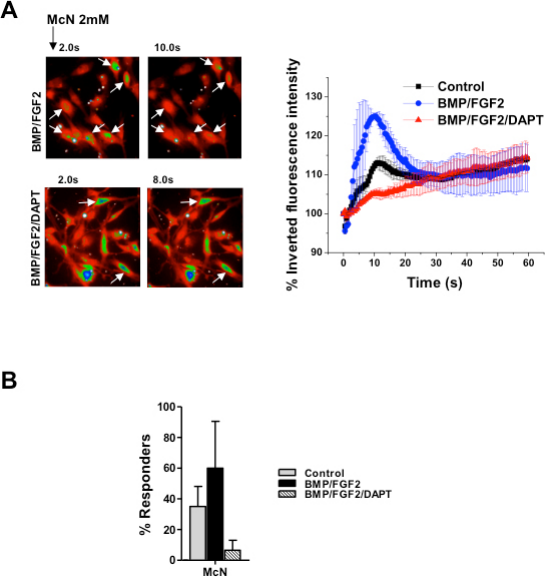
Cytosolic calcium ([Ca^2+^]_i_ ) response to muscarinic receptor activation is consistent with the development of an retinal ganglion cell (RGC) phenotype by Notch-1 inhibition in human Müller glia with stem cell characteristics (hMGSCs). **A**: Examplary heat map images are shown with Fura Red-loaded hMSCs cultured under control conditions and after treatment with basement membrane protein (BMP), basic fibroblast growth factor-2 (FGF2) and N-[N-(3,5-Difluorophenacetyl)-L-alanyl]-S-phenylglycin t-butyl ester (DAPT). The images are recorded at 40× magnification and are representative of fluorescence intensity before (2 s) and at the maximum effect of receptor activation (10 s and 8 s, respectively). Control cells showing a small, slow decrease in fluorescence intensity in response to McN-A343 exposure, which signifies an increase in [Ca^2+^]_i,_ are marked with white arrows. Cells cultured with BMP, FGF2 and DAPT showed a similar response to McN-A343 (left panel). Untreated hMGSCs (control, n=39 cells from three experiments) and hMGSCs treated with BMP, FGF2, and DAPT (n=32 cells from two experiments) responded to the muscarinic receptor agonist McN-A343 (2 mM) with a small reduction in the inverted fluorescence intensity, displayed as a percentage of the value at 0 s, corresponding to a minor rise in [Ca^2+^]_i_, which was greatly augmented after treatment with BMP and FGF2 alone (n=42 cells from two experiments, *p<0.05 and ***p<0.001, respectively). **B**: There was no significant alteration in the percentage of cells responding to the muscarinic receptor agonist McN-A343 with or without differentiation by Notch-1 inhibition (n=50 from four experiments).

Similarly, we investigated the effect of the muscarinic receptor agonist McN-A343 (2 mmoles/l) on [Ca^2+^]_i_ in undifferentiated and differentiated hMGSCs. Figure 4A shows that undifferentiated MIO-M1 cells responded to muscarinic receptor activation with a small transient rise in [Ca^2+^]_i_. This response was substantially augmented following treatment with BMP and FGF2 alone (*p<0.05), but in comparison to control cells, remained unaltered in the in the presence of BMP, FGF2 and DAPT (p>0.05). Conversely, Figure 4B illustrates that the proportion of undifferentiated or differentiated cells, which responded to treatment with McN-A343 with an augmentation of [Ca^2+^]_i_, was not statistically affected (p>0.05).

### Effect of Notch-1 inhibition in hMGSCs on gene expression of nicotinic acetylcholine receptor subunits

**Figure 5:**
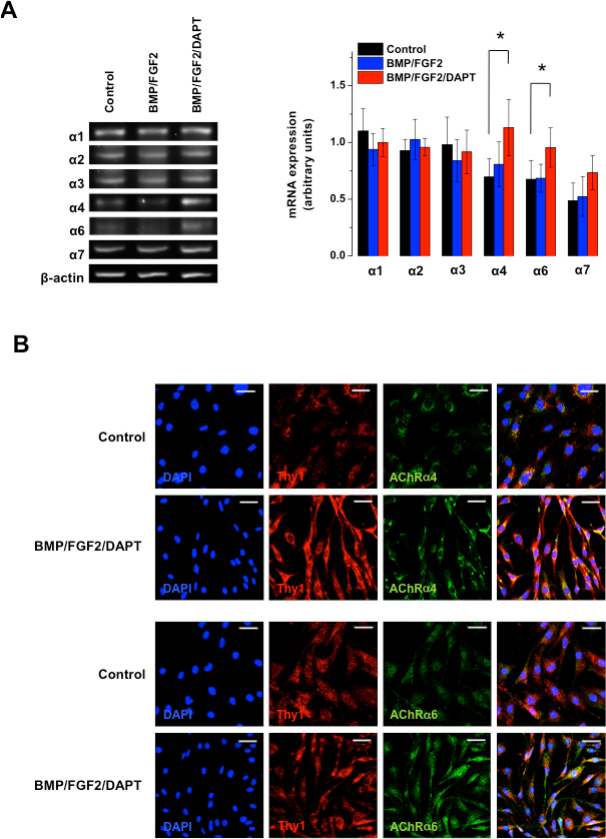
Notch-1 inhibition leads to upregulation of nicotinic α4- and α6-receptor subunits in human Müller glia with stem cell characteristics (hMGSCs). **A**: Exemplary images show mRNA expression of nicotinic acetylcholine receptors (nAChR) α1–4, 6, and 7 in hMGSCs under control conditions, after differentiation with basement membrane protein (BMP) and basic fibroblast growth factor-2 (FGF2) alone or after addition of N-[N-(3,5-Difluorophenacetyl)-L-alanyl]-S-phenylglycin t-butyl ester (DAPT; left panel). Following differentiation of hMGSCs with the Notch-1 inhibitors BMP, FGF2, and DAPT (red bars), mRNA expression of the α4 and α6 nicotinic acetylcholine receptors (nAChR) subunits was significantly upregulated in comparison to untreated cells (control, black bars) or after treatment with BMP and FGF2 alone (blue bars), as shown by reverse transcription PCR (RT-PCR; *p<0.05, n=6, right panel). **B**: Immunohistochemical staining showed an increase in protein expression of the α4 and α6 nicotinic AChR subunits (green) with concomitant upregulation of THY1 by hMGSCs treated with BMP, FGF2, and DAPT, in comparison to control (40× magnification).

The [Ca^2+^]_i_ responsiveness to nicotine has previously been shown to be greatly augmented in hMGSCs after differentiating treatment with BMP, FGF2, and DAPT [13]. As demonstrated in Figure 5A, in undifferentiated hMGSCs mRNA for the nAChR subunits α1–4, 6, and 7 was detected in low quantities. After blocking Notch-1 activity with BMP, FGF2, and DAPT treatment, mRNA for the α4 and α6 nAChR subunits was demonstrated by conventional RT-PCR to be significantly upregulated in comparison to untreated hMGSCs (*p<0.05, n=6). Consistently, Figure 5B shows increased immunocytochemical staining for the α4 and α6 nAChR subunits, concomitantly with upregulation of the RGC marker THY1, in RGC-committed precursors.

### Modulation of the increased [Ca^2+^]_i_ responsiveness to nicotine stimulation in differentiated hMGSCs by pharmacological inhibitors

**Figure 6:**
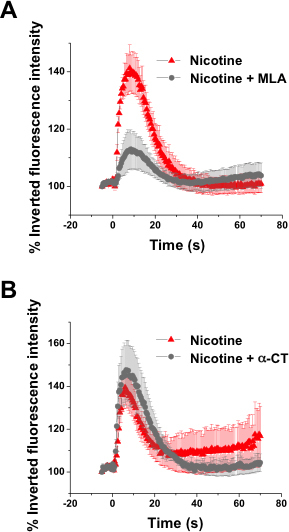
Increased cytosolic calcium ([Ca^2+^]_i_ ) in response to nicotinic receptor activation is greatly attenuated by methyllycaconitine, but not α-conotoxin MII in retinal ganglion cell (RGC)-committed precursors. The effect of pharmacological inhibition of the α4β2 and α6β2 nicotinic acetylcholine receptors (AChRs) with methyllycaconitine (MLA, 100 nmoles/l) and of the α3β2 nicotinic AChR with α-conotoxin MII (α-CT, 100 nmoles/l) on the rise in [Ca^2+^]_i_ triggered by nicotine (2 mM) was assessed. **A**: Methyllycaconitine caused a substantial reduction in the [Ca^2+^]_i_ rise (n=99 cells from 3 experiments) in comparison to stimulation with nicotine alone (n=113 cells from 3 experiments , ***p<0.001). **B**: α-conotoxin MII did not significantly affect the rise in [Ca^2+^]_i_ after stimulation with nicotine (n=254 cells from 4 experiments) in comparison to stimulation with nicotine alone (n=290 cells from 4 experiments).

The [Ca^2+^]_i_ responsiveness to nicotine was confirmed by inhibition of nicotinic receptors with pharmacological antagonists. To demonstrate that upregulated nAChR subunits form functional ion channels and that an increase in [Ca^2+^]_i_ responsiveness to nicotine occurred following Notch-1 inhibition with BMP, FGF2, and DAPT treatment, we tested the effects of a pharmacological inhibitor of the nAChR α3β2, α-conotoxin MII (α-CT, 100 nmoles/l), and methyllycaconitine (MLA, 100 nmoles/l), which blocks α4β2, α6β2, and α7β2 nAChR activation. Figure 6 demonstrates that the [Ca^2+^]_i_ responsiveness to nicotine after Notch-1 inhibition was greatly attenuated by methyllycaconitine to a level similar to untreated hMGSCs (***p<0.001 n=3), while α-conotoxin MII had no significant effect on [Ca^2+^]_i_ at the concentration used (n=4).

## Discussion

The human retina is not known to regenerate or repair after disease or injury. However, it harbors a population of Müller glia with stem cell characteristics, similar to those observed in lower vertebrates, which have been demonstrated to repair and regenerate after retinal damage [5,12]. In vitro*,* hMGSCs have recently been shown to differentiate toward RGCs, the cell type predominantly damaged in glaucoma [13]. We have previously reported that intravitreal transplantation of cells with RGC phenotype in rats, depleted of RGCs by NMDA injection, are capable of partially restoring RGC function [13]. Although this effect may to some extent be ascribed to neurotrophic factors, it is possible that it can also be attributed to the neural function of the transplanted cells. Transplantation success may be affected by cell-fate determination and the ontogenetic stage of the graft [28]. It is therefore vital that the phenotype of potential candidates for cell transplantation be thoroughly investigated. In the present study, we have investigated the functional characteristics, as well as the ontogenetic stage, of hMGSC-derived RGCs.

hMGSCs express high levels of Notch-1 [5], which has been shown in developmental studies to maintain the progenicity of neural stem cells [29]. Since generation of RGCs requires the cessation of Notch-1 activity in the embryonic retina [14,30], we have used Notch-1 inhibition to differentiate hMGSCs toward RGCs, as previously reported [13]. To confirm that we were examining an RGC population, we examined the expression of RGC markers following Notch-1 inhibition. As previously noted, the expression of its downstream effector *HES1* was markedly downregulated [30], while the early RGC marker BRN3B was greatly increased. Following Notch-1 inhibition, the expression of the RGC markers ISL-1, THY-1, and βIII-tubulin were greatly augmented with concomitant downregulation of vimentin and CRALBP. These findings substantiate our previous reports that cells expressing neural morphology and RGC markers can be generated from hMGSCs by Notch-1 inhibition [13].

We subsequently studied the functional properties of hMGSC-derived retinal cells committed to an RGC fate by investigating their [Ca^2+^]_i_ responsiveness to various key neurotransmitters, as well as changes in their expression of neurotransmitter receptors. Although it was initially thought that only neurons contained functional neurotransmitter receptors, it is now generally accepted that while some receptors are exclusively expressed by mature neurons [20], other cell types in the central nervous system, including glial cells, can also respond to certain neurotransmitters [31-33]. In addition, the expression of neurotransmitter receptors in neural precursors varies depending on their developmental stage [16,17]. Müller glial cells and retinal neurons respond to certain neurotransmitters, and these cell types can nevertheless be distinguished by selective agonists, which activate distinct neurotransmitter receptors. Functional expression of nicotinic, NMDA-sensitive glutamatergic, and muscarinic receptors were investigated in this study, since these are differentially expressed in Müller glial cells, RGC precursors, and differentiated RGCs.

While low expression of NMDA receptor subunits has been reported in early retinal development [20,34], this is greatly upregulated during the late stages of retinal development or in the early postnatal period. Concomitantly, a functional response of RGCs and their precursors to the stimulation of glutamatergic receptors with NMDA has been reported to emerge [19,35-37]. In addition, NMDA receptors are highly expressed by mature RGCs [24], as well as by Müller glia [25]. In the present study, we have shown that the [Ca^2+^]_i_ responsiveness to the glutamatergic neurotransmitter receptor agonist NMDA is greatly diminished in hMGSCs following Notch-1 inhibition. This change in sensitivity to NMDA receptor activation after treatment with BMP and FGF2 alone or in combination with the γ-secretase inhibitor DAPT is consistent with hMGSCs acquiring a functional phenotype akin to RGCs. Combined treatment of BMP and FGF2 with DAPT generated cells with RGC phenotype, as this has been shown to promote the expression of molecular markers of newly born RGCs, such as BRN3B.

Muscarinic acetylcholine receptors are expressed at a low level in undifferentiated retinal progenitor cells during early embryogenesis, although they are upregulated during later stages of retinal development [20]. Electrophysiological studies have provided evidence that these receptors form functional ion channels in retinal progenitors and can facilitate an increase in [Ca^2+^]_i_ in response to muscarinic receptor stimulation [17,38,39]. Conversely, Müller glia display low sensitivity to muscarinic receptor agonists [26]. In the present study, partial inhibition of Notch-1 activity with BMP and FGF2 caused a significant increase in the [Ca^2+^]_i_ response to muscarinic receptor stimulation, which suggests a change in the phenotype toward RGCs. On the other hand, differentiation of hMGSCs with a combination of BMP, FGF2, and DAPT, which leads to attenuation of Notch-1 activity [13], resulted in no significant augmentation in the [Ca^2+^]_i_ response to McN-A343 in comparison to control cells. This finding is consistent with the hypothesis that complete Notch-1 inhibition with BMP, FGF2, and DAPT promotes the development of an earlier retinal progenitor phenotype, which does not express functional muscarinic receptors and may be capable of forming RGC progenitors.

Although mRNA expression of the downstream effector of Notch-1, HES1, was markedly attenuated by treatment with BMP and FGF2 alone or in combination with DAPT, this was not reflected by the different patterns of muscarinic receptor responsiveness in these cells. In fact, while treatment with BMP and FGF2 significantly increased the [Ca^2+^]i responsiveness to muscarinic receptor activation, this was attenuated by Notch-1 inhibition with BMP, FGF2, and DAPT. Although beyond the scope of the present study, it may be speculated that other downstream effectors of Notch-1, such as HES5 [40], may be differentially affected by Notch-1 inhibition with BMP, FGF2, and DAPT and contribute to the differentiation of Müller glia cells with stem cell characteristics toward RGCs.

One of the hallmarks of neurons is their responsiveness to nicotinic receptor activation [41], which is characteristic of differentiated RGCs [42], but not undifferentiated precursors committed to an RGC fate [38]. Although Müller glia cells have been reported to express nAChR subunits [43] and respond to stimulation with increased [Ca^2+^]_i_, their sensitivity appears to be much lower than in retinal neurons [26]. During development, RGC expression of nicotinic receptor subunits is first detected in early differentiating RGCs [44]. In the present study, undifferentiated hMSCs and those treated with BMP and FGF2 alone remained largely unresponsive to the stimulation of nAChRs. However, following attenuation of Notch-1 activity with BMP, FGF2 and the γ-secretase inhibitor DAPT, stimulation of nAChRs with nicotine elicited a profound increase in [Ca^2+^]_i_, which may be ascribed to increased expression of the α4 and α6 nicotinic receptor subunits, as indicated by the increase in gene and protein expression observed in this study. Our findings were further substantiated by pharmacological intervention with the α4, α6 and α7 nicotinic receptor blocker methyllycaconitine, which greatly attenuated the [Ca^2+^]_i_ responsiveness of differentiated MIO-M1 cells to nicotine. Since mRNA levels for the α7 nicotinic receptor subunit were not shown to be significantly altered, it is likely that the augmentation of [Ca^2+^]_i_ by nicotine in differentiated hMSCs was largely mediated by the increased levels of α4 and α6 receptors. In addition, the α3β2 blocker α-conotoxin MII did not significant alter the rise in [Ca^2+^]_i_ in response to nicotine, demonstrating the selectivity of its attenuation by methyllycaconitine.

Although Notch-1 inhibition with BMP, FGF2, and DAPT altered the [Ca^2+^]_i_ responsiveness of hMGSCs toward a phenotype consistent with postmitotic RGCs, not all cells appeared to respond equally to neurotransmitter stimulation. This likely reflects the generation of a heterogenous cell population, which may also contain early and late retinal progenitors, although the predominant cell type obtained was functionally similar to RGCs.

Taken together, the results of our study strongly suggest that hMGSCs differentiated with BMP, FGF2, and DAPT generate functional newly born RGCs, a developmental stage that may be advantageous for successful engraftment [28]. The present study therefore provides important evidence that hMGSCs can be differentiated in vitro into functional RGCs, with potential application in cell-based therapies for treating conditions in which RGCs are compromised.

## References

[1] Coles BL, Angenieux B, Inoue T, Del Rio-Tsonis K, Spence JR, McInnes RR, Arsenijevic Y, van der Kooy D (2004). Facile isolation and the characterization of human retinal stem cells.. Proc Natl Acad Sci USA.

[2] Tropepe V, Coles BL, Chiasson BJ, Horsford DJ, Elia AJ, McInnes RR, van der Kooy D (2000). Retinal stem cells in the adult mammalian eye.. Science.

[3] Gu P, Harwood LJ, Zhang X, Wylie M, Curry WJ, Cogliati T (2007). Isolation of retinal progenitor and stem cells from the porcine eye.. Mol Vis.

[4] MacNeil A, Pearson RA, MacLaren RE, Smith AJ, Sowden JC, Ali RR (2007). Comparative analysis of progenitor cells isolated from the iris, pars plana, and ciliary body of the adult porcine eye.. Stem Cells.

[5] Lawrence JM, Singhal S, Bhatia B, Keegan DJ, Reh TA, Luthert PJ, Khaw PT, Limb GA (2007). MIO-M1 cells and similar Müller glial cell lines derived from adult human retina exhibit neural stem cell characteristics.. Stem Cells.

[6] Fischer AJ, Reh TA (2001). Muller glia are a potential source of neural regeneration in the postnatal chicken retina.. Nat Neurosci.

[7] Ooto S, Akagi T, Kageyama R, Akita J, Mandai M, Honda Y, Takahashi M (2004). Potential for neural regeneration after neurotoxic injury in the adult mammalian retina.. Proc Natl Acad Sci USA.

[8] Raymond PA, Barthel LK, Bernardos RL, Perkowski JJ (2006). Molecular characterization of retinal stem cells and their niches in adult zebrafish.. BMC Dev Biol.

[9] Fausett BV, Goldman D (2006). A role for alpha1 tubulin-expressing Müller glia in regeneration of the injured zebrafish retina.. J Neurosci.

[10] Yurco P, Cameron DA (2005). Responses of Müller glia to retinal injury in adult zebrafish.. Vision Res.

[11] Bhatia B, Singhal S, Lawrence JM, Khaw PT, Limb GA (2009). Distribution of Müller stem cells within the neural retina: evidence for the existence of a ciliary margin-like zone in the adult human eye.. Exp Eye Res.

[12] Limb GA, Salt TE, Munro PM, Moss SE, Khaw PT (2002). In vitro characterization of a spontaneously immortalized human Müller cell line (MIO-M1).. Invest Ophthalmol Vis Sci.

[13] Singhal S, Bhatia B, Jayaram H, Becker S, Jones MF, Cottrill PB, Khaw PT, Salt TE, Limb GA (2012). Human Müller Glia with Stem Cell Characteristics Differentiate into Retinal Ganglion Cell (RGC) Precursors In Vitro and Partially Restore RGC Function In Vivo Following Transplantation.. Stem Cells Translational Med..

[14] Austin CP, Feldman DE, Ida JA, Cepko CL (1995). Vertebrate retinal ganglion cells are selected from competent progenitors by the action of Notch.. Development.

[15] Ghai K, Zelinka C, Fischer AJ (2010). Notch signaling influences neuroprotective and proliferative properties of mature Müller glia.. J Neurosci.

[16] Schipke CG, Ohlemeyer C, Matyash M, Nolte C, Kettenmann H, Kirchhoff F (2001). Astrocytes of the mouse neocortex express functional N-methyl-D-aspartate receptors.. FASEB J.

[17] Cai J, Cheng A, Luo Y, Lu C, Mattson MP, Rao MS, Furukawa K (2004). Membrane properties of rat embryonic multipotent neural stem cells.. J Neurochem.

[18] Wong RO (1998). Calcium imaging and multielectrode recordings of global patterns of activity in the developing nervous system.. Histochem J.

[19] Wong RO (1995). Effects of glutamate and its analogs on intracellular calcium levels in the developing retina.. Vis Neurosci.

[20] Das AV, Edakkot S, Thoreson WB, James J, Bhattacharya S, Ahmad I (2005). Membrane properties of retinal stem cells/progenitors.. Prog Retin Eye Res.

[21] Martins RA, Pearson RA (2008). Control of cell proliferation by neurotransmitters in the developing vertebrate retina.. Brain Res.

[22] Lecchi M, McIntosh JM, Bertrand S, Safran AB, Bertrand D (2005). Functional properties of neuronal nicotinic acetylcholine receptors in the chick retina during development.. Eur J Neurosci.

[23] Zhou ZJ, Zhao D (2000). Coordinated transitions in neurotransmitter systems for the initiation and propagation of spontaneous retinal waves.. J Neurosci.

[24] Shen Y, Liu XL, Yang XL (2006). N-methyl-D-aspartate receptors in the retina.. Mol Neurobiol.

[25] Puro DG, Yuan JP, Sucher NJ (1996). Activation of NMDA receptor-channels in human retinal Müller glial cells inhibits inward-rectifying potassium currents.. Vis Neurosci.

[26] Wakakura M, Utsunomiya-Kawasaki I, Ishikawa S (1998). Rapid increase in cytosolic calcium ion concentration mediated by acetylcholine receptors in cultured retinal neurons and Müller cells.. Graefes Arch Clin Exp Ophthalmol.

[27] López T, Lopez-Colome AM, Ortega A (1997). NMDA receptors in cultured radial glia.. FEBS Lett.

[28] MacLaren RE, Pearson RA, MacNeil A, Douglas RH, Salt TE, Akimoto M, Swaroop A, Sowden JC, Ali RR (2006). Retinal repair by transplantation of photoreceptor precursors.. Nature.

[29] Andreazzoli M (2009). Molecular regulation of vertebrate retina cell fate.. Birth Defects Res C Embryo Today.

[30] Nelson BR, Gumuscu B, Hartman BH, Reh TA (2006). Notch activity is downregulated just prior to retinal ganglion cell differentiation.. Dev Neurosci.

[31] Verkhratsky A, Orkand RK, Kettenmann H (1998). Glial calcium: homeostasis and signaling function.. Physiol Rev.

[32] Gallo V, Ghiani CA (2000). Glutamate receptors in glia: new cells, new inputs and new functions.. Trends Pharmacol Sci.

[33] Hósli L, Hosli E, Della Briotta G, Quadri L, Heuss L (1988). Action of acetylcholine, muscarine, nicotine and antagonists on the membrane potential of astrocytes in cultured rat brainstem and spinal cord.. Neurosci Lett.

[34] Martins RA, Linden R, Dyer MA (2006). Glutamate regulates retinal progenitors cells proliferation during development.. Eur J Neurosci.

[35] Sugioka M, Fukuda Y, Yamashita M (1998). Development of glutamate-induced intracellular Ca2+ rise in the embryonic chick retina.. J Neurobiol.

[36] Acosta ML, Chua J, Kalloniatis M (2007). Functional activation of glutamate ionotropic receptors in the developing mouse retina.. J Comp Neurol.

[37] Guenther E, Schmid S, Wheeler-Schilling T, Albach G, Grunder T, Fauser S, Kohler K (2004). Developmental plasticity of NMDA receptor function in the retina and the influence of light.. FASEB J.

[38] Pearson R, Catsicas M, Becker D, Mobbs P (2002). Purinergic and muscarinic modulation of the cell cycle and calcium signaling in the chick retinal ventricular zone.. J Neurosci.

[39] Sakaki Y, Fukuda Y, Yamashita M (1996). Muscarinic and purinergic Ca2+ mobilizations in the neural retina of early embryonic chick.. Int J Dev Neurosci.

[40] Kageyama R, Ohtsuka T (1999). The Notch-Hes pathway in mammalian neural development.. Cell Res.

[41] Dajas-Bailador F, Wonnacott S (2004). Nicotinic acetylcholine receptors and the regulation of neuronal signalling.. Trends Pharmacol Sci.

[42] Lipton SA, Tauck DL (1987). Voltage-dependent conductances of solitary ganglion cells dissociated from the rat retina.. J Physiol.

[43] Kubrusly RC, da Cunha MC, Reis RA, Soares H, Ventura AL, Kurtenbach E, de Mello MC, de Mello FG (2005). Expression of functional receptors and transmitter enzymes in cultured Muller cells.. Brain Res.

[44] Matter JM, Matter-Sadzinski L, Ballivet M (1995). Activity of the beta 3 nicotinic receptor promoter is a marker of neuron fate determination during retina development.. J Neurosci.

[45] Lam DC, Girard L, Ramirez R, Chau WS, Suen WS, Sheridan S, Tin VP, Chung LP, Wong MP, Shay JW, Gazdar AF, Lam WK, Minna JD (2007). Expression of nicotinic acetylcholine receptor subunit genes in non-small-cell lung cancer reveals differences between smokers and nonsmokers.. Cancer Res.

[46] Zarghooni S, Wunsch J, Bodenbenner M, Bruggmann D, Grando SA, Schwantes U, Wess J, Kummer W, Lips KS (2007). Expression of muscarinic and nicotinic acetylcholine receptors in the mouse urothelium.. Life Sci.

[47] Plummer HK, Dhar M, Schuller HM (2005). Expression of the alpha7 nicotinic acetylcholine receptor in human lung cells.. Respir Res.

[48] Liu S, Li J, Tan DT, Beuerman RW (2007). Expression and function of muscarinic receptor subtypes on human cornea and conjunctiva.. Invest Ophthalmol Vis Sci.

